# Prospective associations of psychosocial adversity in childhood with risk factors for cardiovascular disease in adulthood: the MRC National Survey of Health and Development

**DOI:** 10.1186/s12939-017-0656-1

**Published:** 2017-09-07

**Authors:** Emma L. Anderson, Rishi Caleyachetty, Mai Stafford, Diana Kuh, Rebecca Hardy, Debbie A. Lawlor, Abigail Fraser, Laura D. Howe

**Affiliations:** 1 MRC Integrative Epidemiology Unit at the University of Bristol, Bristol, UK; 2 School of Social and Community Medicine, University of Bristol, Oakfield House, Oakfield Grove, Bristol, BS8 2BN UK; 3 The Institute of Applied Health Research, University of Birmingham, Birmingham, UK; 4 MRC Unit for Lifelong Health and Ageing, University College London, London, UK

**Keywords:** Psychosocial, Adversity, Childhood, Cardiovascular disease

## Abstract

**Background:**

Studies assessing associations of childhood psychosocial adversity (e.g. sexual abuse, physical neglect, parental death), as opposed to socioeconomic adversity, with cardiovascular disease (CVD) risk factors in adulthood are scarce. We aimed to assess associations of various forms of psychosocial adversity and cumulative adversity in childhood, with multiple CVD risk factors in mid-life.

**Methods:**

Participants were from the MRC National Survey of Health and Development. Childhood psychosocial risk factors were reported prospectively by parents from 1950-1957, and retrospectively by participants at mean age 43 years in 1989. CVD risk factors were assessed at mean age 60-64 years in 2006-2011. Associations of a summary score of total psychosocial adversity and CVD risk in adulthood were assessed.

**Results:**

There was no consistent evidence that cumulative psychosocial adversity, nor any specific form of psychosocial adversity in childhood, was associated with CVD risk factors in late adulthood. There was some evidence that parental death in the first 15 years was associated with higher SBP (Beta: 0.23, 95% confidence interval: 0.06 to 0.40, *P*=0.01) and DBP (Beta: 0.15, 95% confidence interval: -0.01 to 0.32, *P*=0.07).

**Conclusions:**

We found no evidence that exposure to greater psychosocial adversity, or specific forms of psychosocial adversity during childhood is associated with adult CVD risk factors. Further large population studies are needed to clarify whether parental death is associated with higher systolic and diastolic blood pressure.

**Electronic supplementary material:**

The online version of this article (10.1186/s12939-017-0656-1) contains supplementary material, which is available to authorized users.

## Background

Socioeconomic adversity during childhood (such as low head of household social class, low income, household overcrowding and low parental education) is now recognised as an important risk factor for cardiovascular disease (CVD) [1]. However, consequences of psychosocial adversity in childhood (for example, sexual abuse, emotional and physical neglect) on cardiovascular risk are less clear.

Existing studies have provided some evidence for adverse cardiovascular consequences of specific forms of psychosocial adversity; usually sexual or physical abuse [2–5]. However, very few studies have examined whether or not there is a cumulative effect of multiple adverse exposures in childhood on cardiovascular risk [6–10]. Assessing a cumulative effect of multiple forms of adversity acknowledges that adverse exposures tend to co-occur and that exposure to multiple adverse experiences may have greater adverse effects on health than exposure to only one. Those studies that have assessed a cumulative effect have either considered a very small number of adverse experiences, which are not a comprehensive measure of total adversity [6], or have assessed few cardiovascular outcomes [8, 10], making it difficult to establish the impact on overall CVD risk. Furthermore, there is a lack of studies with prospective data on adverse experiences in childhood that have followed-up participants into older age. No existing studies have attempted to examine possible pathways of association between psychosocial adversity and CVD risk (for example, does greater psychosocial adversity during childhood increase risk of smoking as an adult, or increase body mass index, which in turn increases CVD risk?). There is some evidence to suggest that associations between childhood psychosocial adversity and CVD risk in adulthood may differ between people who have high compared to low socioeconomic position (SEP) in adulthood [7].

In 2,230 men and women from the Medical Research Council National Survey of Health and Development (NSHD), we aimed to assess associations of prospectively-assessed adverse childhood experiences (including maltreatment [abuse or neglect of any kind], sub-optimal maternal bonding, parental physical illness, parental mental illness, parental absence from the household, parental divorce or separation and death of mother or father in childhood) with multiple CVD risk factors in late adulthood (body mass index [BMI], waist circumference, systolic and diastolic blood pressure [SBP, DBP], plasma glucose, insulin, triglycerides, low density lipoprotein cholesterol [LDL] and high density lipoprotein [HDL] cholesterol, C-reactive protein [CRP], carotid intima media thickness [cIMT] and pulse wave velocity [PWV]). We also examined associations of cumulative psychosocial adversity in childhood with these adult CVD risk factors. In secondary analyses, we examined whether these associations differed by adult socioeconomic position (SEP; manual compared to non-manual) and assessed potential mediation by BMI and smoking in adulthood.

## Methods

NSHD is a population-based, social class stratified sample of 5,362 births, of all singleton births that occurred within marriage in a week in March 1946 in England, Scotland and Wales. Between 2006 and 2011, the NSHD scientific and data collection team invited all 2856 eligible study members (aged 60–64 years) who were known to be alive and have an address in England, Scotland, or Wales for assessment at 1 of 6 clinical research facilities or by a research nurse at home. A total of 2230 (78%) were assessed with 1690 (59.2%) attending a clinic and the remaining 539 seen at home [11]. Ethical approval for the study was obtained from the Greater Manchester Local Research Ethics Committee and the Scotland A Research Ethics Committee.

### Assessing cardiovascular risk factors

BMI, waist circumference, systolic and diastolic blood pressure, plasma glucose, insulin, triglycerides, LDL cholesterol, HDL cholesterol, CRP, cIMT and PWV were assessed at clinical research facilities, or during a home visit from a research nurse, between the ages of 60–64 years. cIMT and PWV were only measured in participants attending the clinics. Methods of assessment of each CVD risk factor are described in the Additional file 1.

### Adverse experiences in childhood

The following types of psychosocial adversity were included in our analyses: maltreatment [abuse or neglect of any kind], sub-optimal maternal bonding, parental physical illness, parental mental illness, parental absence from the household, parental divorce or separation and death of mother or father in childhood. At age 43 years, maltreatment was assessed retrospectively by asking participants ‘as a child do you feel you were mistreated by your parents in any way?’. Parental bonding was also assessed retrospectively at age 43 years with the *Parental Bonding Instrument* (PBI) [12]. During a health visitor interview mothers reported if they or their partner had a serious physical or psychiatric illness during the first 15 years of their child's life. At age 6, mothers were asked about the longest amount of time they had ever been separated from their children. Experience of parental divorce or death before age 16 years was recorded.

### Assessment of covariables

Participants SEP in childhood was assessed using their father’s occupation, which was reported prospectively at a health visitor interview at age 4 years (if information was missing at age 4, reports at 7 or 11 years were used). Occupations were used to allocate head of household social class groups according to the British Registrar General’s Social Classification. Participants own SEP in adulthood was assessed using their own and their partner’s current occupation at age 53. The highest level of occupation was used to allocate head of household social class groups using the British Registrar General’s Social Classification. Childhood and adulthood SEP were both coded as ‘high’ (professional, managerial and technical occupations) or ‘low’ (including skilled, partly skilled and unskilled occupations). Smoking was assessed at age 60-64 years by questionnaire and coded as current, ex- or never smoker. Age was recorded at the time of CVD risk factor measurements along with details of current medication use.

## Statistical analysis

Distributions of insulin, triglyceride and CRP were right skewed, therefore the log of these variables were used in analysis to ensure that model residuals were approximately normally distributed. Regression coefficients were back transformed so that the results for these variables are ratios of geometric means per unit increase in the exposure.

### Cumulative psychosocial adversity

Our *a priori* preferred analysis strategy was to create a weighted score of cumulative psychosocial adversity using confirmatory factor analysis. However, this approach was not successful; model fit was very poor and factor loadings for all psychosocial adversity variables except for parental bonding indices and maltreatment were low (≤0.2). Modifying the second order factor model to exclude those indicators with poor factor loadings and high residual variances would leave only parental bonding indices and maltreatment, thus the score would not capture cumulative psychosocial adversity. It was therefore decided that we would not proceed with the latent cumulative psychosocial adversity score. Full methods for the factor analysis and details of the model fit and factor loadings are provided in the Additional file 1.

We present instead results using a summary score of all measured psychosocial adversity indicators, as has been used in previous studies of the health consequences of early life adversity [13]. All adversity variables were binary (coded as ‘0’ for not exposed and ‘1’ for exposed) except for maternal lack of care and overprotection, which were both continuous scores [14]. Thus, a binary variable was derived using validated cut-points [14] to indicate sub-optimal maternal bonding maternal lack of care and overprotection. All binary variables were then summed to produce a summary score of the number of adverse experiences each participant was exposed to during childhood (ranging from 0-7). The summary score was categorised as 0, 1, 2, 3+. Linearity of associations between the summary score and CVD risk factors was assessed using a likelihood ratio test to compare models with the score as a continuous variable to models with the score as a categorical variable with indicators. There was no evidence of a threshold effect (results available on request). Thus, the categorical summary score was included as a linear term and results are interpreted as a unit change in the outcome per one category increase in the number of adverse experiences (i.e. from 0 to 1, 1 to 2, and 2 to 3+). Associations of the summary score with the CVD risk factors were estimated in Stata MP version 14, using multiple linear regression in the following models: (1) adjusted for age at outcome assessment (2) additionally adjusted for childhood SEP (3) additionally adjusted for potential mediation by adult SEP and (4) additionally adjusted for potential mediation by adult BMI and smoking.

### Additional analyses

We tested for an interaction between the summary score and adult SEP, using likelihood ratio tests to compare models with and without the interaction term for score by adult SEP included. Sex interactions were assessed with likelihood tests comparing models with and without a score by sex interaction term. We also assessed whether results were similar when (i) adjusting models with lipids, blood pressure and insulin and glucose for current use of statin, antihypertensive and diabetic medication, respectively and (ii) removing participants that reported taking any of these medications at the time of outcome assessment.

#### Eligibility criteria and missing data

Participants were included in the study if they had at least one measure of psychosocial adversity in childhood and at least one cardiovascular outcome (*n*=2230). To minimise selection bias and increase efficiency, multivariate multiple imputation was used to impute missing data for eligible participants under a missing at random assumption. Full details of the multiple imputation procedure are in the Additional file 1. As a sensitivity analysis, analyses were repeated on the subgroup with no missing data for any variable (complete case).

## Results

Table 1 compares characteristics of participants included in the current study to those who were excluded due to missing data due to death, living abroad, withdrawing or being lost to the study. On average, there was a lower prevalence of maltreatment, low maternal bonding and parental physical illness or disability among included compared to excluded participants. Included participants also had a lower proportion not exposed to any form of psychosocial adversity in childhood, fewer never-smokers or ex-smokers and a lower proportion of manual childhood social class and low adult social class. Correlations between different adverse experiences were weak (0.002 to 0.29, Additional file 1: Table S2). Prevalence of psychosocial adversity exposures was similar among participant with manual compared with non-manual SEP, except for parental physical illness which was higher in the manual SEP subgroup (Additional file 1: Table S3).

**Table 1:** Distribution of exposures and outcomes in participants included in the study compared to those excluded due to missing data

	Included^a^ (*n*=2230)		Excluded^a^ (*n*=3132)		
	Percentage (%)	N with data	Percentage (%)	N with data	*P* value for difference
Psychosocial adversity exposures					
Maltreated	5.5	1998	7.7	1042	0.02
Low maternal bonding	17.7	1952	20.8	992	0.04
Parental absence from household	1.9	2230	2.0	3132	0.80
Parental physical illness or disability	23.8	2230	18.4	3132	<0.01
Parental mental illness	2.2	2230	2.1	3132	0.85
Parental divorce or separation	6.1	2230	5.7	3132	0.48
Parental death	7.8	2230	7.4	3129	0.64
Cumulative adversity score					
0	52.4	1862	64.9	934	
1	35.9		26.8		<0.01
2	9.5		7.2		
3+	2.2		2.2		
Outcomes	Mean (SD)/Median (IQR)	N with data	Mean (SD)/Median (IQR)		*P* value
BMI (kg/m^2^)	27.9 (4.8)	2217	-		-
Waist circumference (cm)	96.5 12.9	2217	-		-
Systolic blood pressure (mm/Hg)	136.4 (18.3)	2216	-		-
Diastolic blood pressure (mm/Hg)	77.8 (10.0)	2216	-		-
Glucose (mmol/dl)	5.7 (1.2)	2009	-		-
Insulin (u/ml)^b^	44 (29, 66)	1038	-		-
Triglycerides (mmol/l)^b^	1.1 (0.8, 1.6)	1981	-		-
LDL cholesterol (mmol/l)	3.5 (1.0)	1969	-		-
HDL cholesterol (mmol/l)	1.6 (0.4)	2064	-		-
CRP (mmol/l)^b^	2.1 (1.3, 3.8)	2063	-		-
CIMT (mm)	0.7 (0.1)	1564	-		-
PWV	8.9 (7.8)	1287	-		-

BMI – body mass index. LDL- low density lipoprotein. HDL – high density lipoprotein. CRP – C-reactive protein. CIMT – carotid intima-media thickness. PWV – pulse wave velocity.

Psychosocial adversity exposures are given as percentages in the exposed group.

^a^Included participants are those who had at least one measure of psychosocial adversity in childhood and at least one cardiovascular risk factor. Characteristics are presented on complete case for each variable and missing data was imputed using multivariate multiple imputation. Excluded participants are those excluded due to missing data and characteristics are presented for those with data available for each variable. There are no available data for cardiovascular risk factors in excluded participants as they did not attend the clinic at age 64 years.

^b^Median and interquartile range are presented for non-normally distributed variable

### Associations of psychosocial adversity in childhood with CVD risk factors in mid-life

#### Cumulative psychosocial adversity

Table 2 shows associations of the psychosocial adversity summary score with CVD risk factors, in the model adjusting for age and childhood SEP. Results from all other models are presented in Additional file 1: Table S4. There was no statistical evidence of a sex interaction with adversity score for any CVD risk factor. There was no evidence that psychosocial adversity was associated with any of the CVD risk factors in the age adjusted or confounder adjusted models. Point estimates remained similar after additional adjustment for potential mediation by adult SEP, adult BMI and smoking at the time of outcome assessment.

**Table 2:** Associations of cumulative psychosocial adversity in childhood and CVD risk factors at mean age 64 years

	Adjusted for age, sex and childhood SEP	
	B (95% CI)	*P*
BMI (kg/m^2^)	0.02 (-0.25, 0.29)	0.88
Waist circumference (cm)	0.14 (-0.53, 0.82)	0.68
SBP (mm/Hg)	0.22 (-0.79, 1.22)	0.67
DBP (mm/Hg)	0.27 (-0.28, 0.82)	0.34
Insulin (u/ml)	1.02 (0.97, 1.06)	0.43
Glucose (mmol/l)^a^	-0.01 (-0.08, 0.06)	0.83
Triglycerides (mmol/l)^a^	1.01 (0.98, 1.05)	0.37
HDL-c (mmol/l)	-0.01 (-0.03, 0.02)	0.64
LDL-c (mmol/l)	-0.03 (-0.09, 0.03)	0.38
CRP (mmol/l)^a^	1.01 (0.96, 1.07)	0.60
CIMT (mm)	0.001 (-0.01, 0.01)	0.55
Pulse wave velocity (m/s)	-0.15 (-0.51, 0.20)	0.40

SEP – socioeconomic position. BMI-body mass index. SBP – systolic blood pressure. DBP – diastolic blood pressure. HDL-c – high density lipoprotein cholesterol. LDL-c - low density lipoprotein cholesterol. CRP – C reactive protein. CIMT – carotid intima-media thickness.

^a^Coefficients for insulin, triglycerides and CRP have been back transformed from the natural log and can be interpreted as a ratio of geometric means, (e.g. a coefficient of 1.02 would be interpreted as an average of 2% increase in the outcome per category increase in cumulative psychosocial adversity)

#### Individual forms of psychosocial adversity

There was no evidence that specific types of psychosocial adversity were associated with any of the CVD risk factors (Additional file 1: Tables S6) except that exposure to parental death during childhood was associated with, on average, 3.2mm/Hg (0.41mm/Hg to 6.0mm/Hg) higher SBP and 1.53 (0mm/Hg to 3.06mm/Hg) higher DBP, adjusted for age at outcome assessment, sex and childhood SEP.

### Additional analyses

Findings from the complete case analysis were generally consistent with those from the imputed data, in that there was no consistent evidence of an association between the summary score for psychosocial adversity and CVD risk factors (Additional file 1: Table S7). However, point estimates were larger (although generally in the same direction) and confidence intervals were wider. Results were also similar after adjusting for antihypertensive, statin or diabetes medication use (Additional file 1: Table S8) and after removing participants who were taking such medications (Additional file 1: Table S9).

There was no statistical evidence that associations differed between participants with low and high adult SEP. However, it is worth noting that six of the twelve estimates of association were in opposite directions in the two adult SEP groups, i.e. increasing cumulative adversity was associated with lower BMI, waist circumference, SBP, DBP, glucose and HDL and higher LDL in participants with high SEP, but with higher BMI, waist circumference, SBP, DBP, glucose and HDL and lower LDL in participants with low adult SEP (Additional file 1: Table S5). That said, there was no evidence that cumulative psychosocial adversity was associated with any of the CVD risk factors in either adult SEP group, as all confidence intervals crossed the null.

## Discussion

We found no consistent evidence to suggest that cumulative psychosocial adversity (as measured by a summary score), or any specific form of psychosocial adversity in childhood, was associated with CVD risk factors in late adulthood. There was some evidence that parental death in the first 15 years was associated with higher SBP and DBP, however, this could be due to a genetic susceptibility in both generations, rather than due to any psychosocial adversity. Given the lack of association with any of the other ten CVD risk factors, this finding needs further replication in other large, well characterised studies. We found no statistical evidence to suggest that associations of cumulative psychosocial adversity in childhood with CVD risk factors differed between participants with low adult SEP compared to high. There was, however, some difference in the direction of point estimates in the two groups, which may suggest a lack of power to detect an interaction. Further studies are needed to clarify whether any effect of psychosocial adversity on CVD risk differs depending on adult SEP.

The summed score method of assessing cumulative psychosocial adversity that we have used in this study assumes that each adverse experience has the same direction and magnitude of association with CVD risk factors, which may be an unrealistic assumption [15]. However, factor analysis, which weights each adversity variable based on correlations with other types of adversity, did not work in this dataset, as described in the methods. Other approaches to weighting the items in a score are also available, e.g. using theory-based weights or weighting by the inverse of the prevalence. For the former option, we did not feel that there was sufficient evidence to predetermine weights for each type of adversity. We decided not to use weights based on the inverse of the prevalence because in this sample the prevalence did not match up to the ‘severity’ of adversity, for example parental death was more common than parental divorce.

There are a number of possible reasons for finding no evidence of associations for both cumulative and specific forms of childhood psychosocial adversity. It is possible that associations may attenuate with age; the cohort were 60-64 years of age at outcome assessment and it is plausible that we may have found evidence of associations if outcomes were assessed earlier. Our study may have lacked power to detect associations due to the sample size, particularly given that the prevalence of some of the adverse exposures was low (e.g. 1.9% maternal absence from the household and 2.2% parental mental illness). Furthermore, loss to follow up bias/survivor bias could potentially have resulted in bias, which we might expect to be towards the null, given the lower prevalence of some psychosocial adversity factors and lower cumulative adversity in the included sample versus those excluded due to missing data. Finally, it is worth noting that these childhood adversity exposures were previously found to be related to the General Health Questionnaire and performance-based physical capability assessed at similar ages to the outcomes in this study [16]. It is worth noting that, although our findings are largely null, they do not remove the need to provide support to those who experience adversity during childhood. Our findings suggest that any interventions put into place are unlikely to influence CVD risk, but that is not to say they would not confer benefits elsewhere (for example, improved mental health outcomes).

### Comparison to other studies

There was evidence in our study that parental death was associated with higher SBP and DBP. However, other studies have reported conflicting findings with one reporting no association between parental death and blood pressure [17] and the other reporting lower blood pressure among those who experienced parental death in childhood [18]. Very few studies have assessed associations of cumulative psychosocial adversity in childhood with multiple CVD risk factors in adulthood. One other study in NSHD assessed cumulative psychosocial adversity in relation to physical capability and the General Health Questionnaire and found that greater psychosocial adversity was associated with poorer physical capability and common affective symptoms in older age [16]. Bleil et al reported adverse family environment and abuse in childhood to be associated with increased CVD risk (based on similar outcomes to our study) in 650 pre-menopausal adult women [9]. One other study assessed associations of multiple forms of psychosocial adversity in childhood with CVD risk in adulthood, and found that accumulation of 4 or more adverse experiences significantly increased BMI, waist circumference and resting heart rate [10]. We observed no association with maltreatment (sexual or non-sexual abuse) in this study. We are not aware of any other studies that have specifically assessed whether associations between cumulative psychosocial adversity and CVD risk differ among people who have a high compared to low adult SEP. However, Halonen et al. did assess childhood adversity and adult neighbourhood disadvantage in relation to hypertension, dyslipidaemia, diabetes mellitus and obesity in 37,699 adults from the Finnish Public Sector Study. They reported that individuals with both childhood psychosocial adversity and adult neighbourhood disadvantage had increased CVD risk, but those with only one of these exposures had little excess risk [7]. Several other studies have reported childhood SEP (as opposed to childhood psychosocial adversity) to be strongly associated with adult CVD risk [1].

### Strengths and limitations

NSHD is a large, well characterised birth cohort, allowing us to assess a number of psychosocial adversity exposures and CVD risk factors. A major strength of this study is that most of the psychosocial adversity exposures were prospectively reported. This means that some of the adversity exposures are less likely to suffer from recall bias compared with retrospectively assessed measures. The main limitation of this study is the potential for bias due to missing data. As with all longitudinal cohort studies, there was inevitably loss to follow-up through death and migration as well as withdrawals. As mentioned above, we might expect this bias to be towards the null in this case, given the lower prevalence of some psychosocial adversity factors (for example maltreatment and parental physical illness or disability) and lower cumulative adversity in those participants included in our study sample versus those excluded due to missing data. To address this limitation, we compared findings across both imputed data, which (provided the data are missing at random) minimises potential for bias and complete case data (with no missing data). Point estimates were broadly similar across the two datasets, albeit with wider confidence intervals in the smaller sample with complete data. As previously stated, the current sample of participants include a larger proportion of ‘high SEP’ participants (based on father’s SEP) than were initially enrolled into NSHD. NSHD was also a social class-stratified sample meaning the prevalence of psychosocial adversity in our sample may not be representative of the general population. There are some limitations to the measures used in our study. Firstly, our measure of social class is based on occupation and does not include specific information on income or other measures of deprivation such as neighbourhood. Secondly, parental physical illness, maltreatment and parental bonding were assessed retrospectively and may be prone to recall bias. That said, there is no gold standard method for collecting data on adverse experiences in childhood and prospective parental or self-reporting during childhood may be equally biased. A review examining the validity of adult retrospective reports of adverse childhood experiences concluded that retrospective recall in adult life of exposure to adverse experiences in childhood is sufficiently valid [19].

## Conclusions

We found no evidence to suggest that greater psychosocial adversity, or exposure to specific forms of psychosocial adversity during childhood is associated with CVD risk factors in adulthood in this birth cohort study. This contrasts with studies showing strong evidence that low childhood social class is associated with greater CVD risk. Further large population studies are needed to clarify whether the effect of cumulative psychosocial adversity on CVD risk is modified by adult SEP, and whether parental death is associated with higher systolic and diastolic blood pressure.

## Additional file

Additional file 1: Supplemental material for CVD risk factors. (DOCX 63 kb)
