# Supplementary material for: Prospective associations of psychosocial adversity in childhood with risk factors for cardiovascular disease in adulthood: the MRC National Survey of Health and Development
Source: Int J Equity Health. 2017 Sep 7;16:170. doi: 10.1186/s12939-017-0656-1 (PMC5590112; doi:10.1186/s12939-017-0656-1)
Supplement: Additional file 1: — Supplemental material for CVD risk factors. (DOCX 63 kb) [file 12939_2017_656_MOESM1_ESM.docx]

Additional file 1

Assessment of cardiovascular risk factors

Height (measured to the nearest 0.1 cm) and weight (to the nearest 0.1 kg) were measured according to standard protocol by trained nurses and BMI was calculated as weight in kilograms divided by height in meters-squared. Waist circumferences were measured to the nearest 1mm according to a standardized protocol. Systolic and diastolic blood pressure (BP) was measured twice using the Omron HEM-705 (Omron Corp., Tokyo, Japan) automated digital oscillometric sphygmomanometer and appropriate cuff sizes, with the participant in a seated position. We used the second reading for the analysis. Overnight fasting blood samples were obtained during home or clinic visits. Aliquots were stored at −80°C and couriered monthly, on dry ice, to the MRC Human Nutrition Research (HNR) laboratory in Cambridge. Plasma insulin was measured the DELFIA^TM^ two-site fluoroimmunometric assay (Perkin-Elmer AutoDelfia analyser. Glucose was measured with enzymatic assays (hexokinase) measured colorimetrically on a Siemens Dimension Xpand analyser. Lipids were measured colorimetrically on a Siemens Dimension Xpand analyser. CRP was measured by particle-enhanced immunoturbidimetric assay on a Siemens Dimension Xpand analyser. The left and right common carotid arteries were imaged longitudinally one centimetre proximal to the carotid bifurcation following a standardized protocol.(1) All scans were performed with the patient in supine position, after 10 minutes of rest. All measures were undertaken using an ultrasound scanner (Vivid I, GE Healthcare) with a high resolution probe (12Mhz). Carotid-femoral Pulse Wave Velocity (PWV) was calculated using pulse pressure waveforms obtained from the carotid and femoral arteries. PWV was measured, using the Vicorder device, by placing a 100mm wide pressure cuff at the upper right thigh and another 30mm partial cuff directly over the right carotid artery. The cuffs were inflated simultaneously to 65mmHg for approximately 10-15 seconds until clear, high quality waveforms (identified by the observer) could be obtained from both arteries. This reading was recorded and the entire process repeated three times to provide 3 PWV measurements. Path length was measured between the cuffs and defined as the distance between the suprasternal notch directly to the top of the femoral cuff (as indicated by the manufacturer). This measurement was input into the Vicorder machine and the transit time and PWV were automatically calculated by an integral algorithm.

**Confirmatory factor analysis**

***Individual forms of psychosocial adversity***

Factor analyses were conducted using Mplus version 7.31 (Muthén & Muthén, 2008). Confirmatory first order factor analyses were used to estimate continuous latent constructs from responses to multiple questions (detailed in Table S1 below) about parental bonding: maternal lack of care and overprotection. Table S1 details the prevalence and the factor loadings for each of the questions asked (i.e. how well each variable loaded onto each latent psychosocial adversity exposure) with larger numbers representing a better loading. Any value above 0.4 was considered to be an acceptable factor loading, although this value is arbitrary. To assess the fit of each first order factor model, three fit statistics were used: (i) root-mean-square error of approximations (RMSEA)(2); (ii) comparative fit index (CFI)(3); and (iii) Tucker– Lewis fit index (TLI).(4) Table S1 below details the model fit statistics for maternal lack of care and overprotection (i.e. first order factors). Factor loadings for most questions were reasonable (>0.4) and model fit for the lack of maternal care factor was also reasonable. However, model fit for the overprotection factor was poor.

***Cumulative psychosocial adversity in childhood***We conducted a second order factor analysis model to capture cumulative psychosocial adversity from the latent lack of maternal care and maternal overprotection constructs, plus observed binary indicators for maltreatment (abuse or neglect of any kind), parental physical illness, parental mental illness, parental absence from the household, parental divorce or separation and death of mother or father in childhood. Table S1 details the prevalence for each of the observed binary indicators. Adverse experiences that are more strongly correlated with other adverse experiences (and therefore experiences that more likely co-occur) are assigned higher factor loadings, and therefore contribute more to the cumulative psychosocial adversity factor score. Table S1 details the factor loadings for the lack of maternal care and overprotection constructs and each of the observed binary indicators, onto the cumulative psychosocial adversity score. Factor loadings for the latent constructs for lack of maternal care and overprotection were reasonable (0.78 and 0.74, respectively). However factor loadings of all other observed binary indicators (except maltreatment) were very low (<0.2). Model fit was poor, suggesting that our measures of psychosocial adversity were not adequate indicators of the latent construct of interest (i.e. cumulative psychosocial adversity). Modifying the second order factor model to exclude those indicators with poor factor loadings and high residual variances would leave only parental bonding indices and maltreatment, thus the score would not be capturing cumulative psychosocial adversity. It was therefore decided that we would not proceed with the latent cumulative psychosocial adversity score.

**Table S1. Prevalence and model fit for psychosocial adversity factors**

| **LATENT ADVERSITY CONSTRUCT**  Exact questions asked in questionnaires | **% Prevalence in included (n=2330)*** | **% Prevalence in excluded (n=3132)**** | **P for difference*** | **Factor loadings for each variable** | **Model fit statistics for each latent construct** |
| --- | --- | --- | --- | --- | --- |
| **First order factors** | | | | | |
| **MATERNAL LACK OF CARE** |  |  |  |  |  |
| Did your mother speak to you in a warm and friendly voice? (RS) | 5.4 | 7.0 | 0.07 | 0.85 | RMSEA = 0.04  CFI =0.99 TLI = 0.99 |
| Did your mother help you as much as you needed? (RS) | 7.8 | 2.3 | 0.69 | 0.89 |  |
| Problems understood by the mother (RS) | 19.1 | 19.0 | 0.94 | 0.89 |  |
| Was your mother affectionate towards you? (RS) | 12.3 | 14.1 | 0.14 | 0.89 |  |
| Did your mother make you feel you were not wanted? | 13.8 | 17.2 | 0.01 | 0.44 |  |
| Did your mother talk things over with you? (RS) | 18.5 | 18.9 | 0.76 | 0.85 |  |
| Did your mother praise you? (RS) | 20.3 | 20.8 | 0.76 | 0.79 |  |
| Did your mother enjoy talking things over with you? (RS) | 30.7 | 30.4 | 0.84 | 0.79 |  |
| Did your mother frequently smile at you? (RS) | 15.1 | 17.9 | 0.05 | 0.76 |  |
| Did your mother seem to understand what you needed or wanted? (RS) | 25.4 | 27.7 | 0.16 | 0.85 |  |
| Did your mother want you to grow up? | 26.6 | 23.8 | 0.09 | 0.21 |  |
| Did your mother make you feel better when you were upset? (RS) | 14.4 | 18.6 | <0.01 | 0.80 |  |
| **MATERNAL OVERPROTECTION** |  |  |  |  |  |
| Did your mother allow you to things you liked doing? (RS) | 10.2 | 12.1 | 0.11 | 0.64 | RMSEA = 0.14  CFI =0.74 TLI = 0.68 |
| Did your mother try to control what you did? | 32.8 | 36.8 | 0.03 | 0.61 |  |
| Did your mother let you decide things for yourself? (RS) | 20.23 | 24.6 | <0.01 | 0.74 |  |
| Did your mother give you the freedom you wanted? (RS) | 34.1 | 35.6 | 0.38 | 0.82 |  |
| Did your mother invade your privacy? | 23.1 | 28.3 | <0.01 | 0.61 |  |
| Did your mother let you go out as often as you wanted? (RS) | 35.9 | 36.5 | 0.74 | 0.78 |  |
| Did you ever feel like you could not look after yourself if your mother was not around? | 20.7 | 25.9 | <0.01 | 0.68 |  |
| Did your mother like you to make your own decisions? (RS) | 17.0 | 20.8 | 0.01 | 0.75 |  |
| Did your mother try to make your dependent on her? | 22.9 | 25.8 | 0.07 | 0.57 |  |
| Was your mother overprotective of you? | 30.0 | 33.1 | 0.08 | 0.60 |  |
| Did your mother tend to baby you? | 25.8 | 32.6 | <0.01 | 0.50 |  |
| Did your mother allow you to dress in any way you pleased? (RS) | 33.5 | 34.7 | 0.50 | 0.55 |  |
| **Second order factor** | | | | | |
| **CUMULATIVE PSYCHOSICIAL ADVERSITY** |  |  |  |  | RMSEA = 0.08  CFI =0.80 TLI =0.78 |
| Lack of care factor | - | - | - | 0.78 |  |
| Overprotection factor | - | - | - | 0.74 |  |
| Parental physical illness | 23.8 | 18.4 | <0.01 | -0.04 |  |
| Parental mental illness | 2.2 | 2.1 | 0.85 | 0.13 |  |
| Maltreated | 5.5 | 7.7 | 0.02 | 0.54 |  |
| Parental absence | 1.9 | 2.0 | 0.80 | 0.05 |  |
| Parental divorce or separation | 6.1 | 5.7 | 0.48 | 0.11 |  |

RS=Reverse Score. All prevalence estimates are given for the ‘Yes’ category, except when RS is indicated, where prevalence is given for the ‘No’ category.
*Prevalence of exposure in participants included in the analysis
**Prevalence of exposure in participants excluded from the analysis due to missing data of either all adversity exposures or all cardiovascular risk factors.

**Multivariate Multiple Imputation Procedure**

Participants included in this study had valid data for at least one measure of psychosocial adversity during childhood and at least one cardiovascular disease (CVD) risk outcome (n=2230). To minimise selection bias and increase efficiency, multivariable multiple imputation using chained equations was used to impute missing data for psychosocial adverse experiences, CVD risk outcomes and potential confounders for eligible participants. Regression switching was used in Stata, as described by Royston.(4) Twenty cycles of regression switching were carried out and 20 imputation datasets were generated.

The multiple multivariable imputation approach creates a specified number of copies of the data (in our case, 20 copies) in which missing values are imputed by chained equations, with an appropriate level of randomness. The main results presented in this paper on the multiple imputation datasets are obtained by averaging the results from each of these 20 datasets using Rubin’s rules. In this procedure, the standard errors for any regression coefficients (used to calculate p-values and 95% confidence intervals) take account of the uncertainty in the imputations as well as uncertainty in the estimate. There was no substantial collinearity between variables included in the multiple imputation models. The analyses based on these multivariable imputations all include data from the 2230 participants. We also repeated analyses including only those participants with complete data on all variables used in our analyses, i.e. with no missing data (n=569). Results from the complete case analyses were broadly similar to those found based on the multiple imputation datasets, except they had wider confidence intervals due to the reduction in sample size.

**Table S2: Pearson’s correlation coefficients of psychosocial adverse experiences in NSHD**

|  | **Maltreatment** | **Sub-optimal maternal bonding** | **Parental absence from household** | **Parental physical illness or disability** | **Parental mental illness** | **Parental divorce or separation** | **Parental death** |
| --- | --- | --- | --- | --- | --- | --- | --- |
| **Maltreatment** | 1.00 |  |  |  |  |  |  |
|  |  |  |  |  |  |  |  |
| **Sub-optimal maternal bonding** | 0.29 | 1.00 |  |  |  |  |  |
|  |  |  |  |  |  |  |  |
| **Parental absence from household** | 0.02 | 0.01 | 1.00 |  |  |  |  |
|  |  |  |  |  |  |  |  |
| **Parental physical illness or disability** | 0.01 | -0.01 | 0.01 | 1.00 |  |  |  |
|  |  |  |  |  |  |  |  |
| **Parental mental illness** | 0.06 | 0.01 | <0.012 | -0.02 | 1.00 |  |  |
|  |  |  |  |  |  |  |  |
| **Parental divorce or separation** | 0.15 | 0.05 | 0.09 | -0.04 | 0.04 | 1.00 |  |
|  |  |  |  |  |  |  |  |
| **Parental death** | 0.01 | 0.01 | 0.16 | <0.014 | 0.04 | -0.03 | 1.00 |

**Table S3. Distribution of adverse psychosocial experiences by childhood SEP**

| **Adverse experiences (%)** | **Non-manual childhood SEP (n=981)** | **Manual childhood SEP (n=1137)** | **P for difference** |
| --- | --- | --- | --- |
| **Maltreatment** | 5.0 | 5.5 | 0.61 |
| **Sub-optimal maternal bonding** | 17.4 | 17.4 | 0.96 |
| **Child physical illness** | 14.8 | 14.5 | 0.86 |
| **Parental absence from household** | 1.7 | 2.1 | 0.53 |
| **Parental physical illness or disability** | 20.2 | 29.0 | <0.01 |
| **Parental mental illness** | 2.1 | 2.4 | 0.72 |
| **Parental divorce or separation** | 4.9 | 6.7 | 0.08 |
| **Parental death** | 6.9 | 7.8 | 0.43 |

SEP – socioeconomic position. Distribution of binary observed variables is presented as a percent prevalence in the exposed group and the P for difference was obtained from a chi^2^ test.

**Table S4. Associations of cumulative psychosocial adversity in childhood and CVD risk factors at mean age 64 years**

|  | **Adjusted for age** | |  | **Adjusted for age and childhood SEP** | |  | **Adjusted for age, childhood SEP and potential mediation by adult SEP** | |  | **Adjusted for age, childhood SEP and potential mediation by adult SEP and BMI** | |
| --- | --- | --- | --- | --- | --- | --- | --- | --- | --- | --- | --- |
|  | **B (95% CI)** | **P** |  | **B (95% CI)** | **P** |  | **B (95% CI)** | **P** |  | **B (95% CI)** | **P** |
| BMI (kg/m^2^) | 0.1 (-0.16, 0.37) | 0.46 |  | 0.02 (-0.25, 0.29) | 0.88 |  | 0.01 (-0.25, 0.28) | 0.92 |  | 0.01 (-0.26, 0.27) | 0.96 |
| Waist circumference (cm) | 0.33 (-0.34, 1.01) | 0.33 |  | 0.14 (-0.53, 0.82) | 0.68 |  | 0.14 (-0.54, 0.81) | 0.69 |  | 0.06 (-0.29, 0.4) | 0.75 |
| SBP (mm/Hg) | 0.37 (-0.63, 1.37) | 0.47 |  | 0.22 (-0.79, 1.22) | 0.67 |  | 0.21 (-0.8, 1.21) | 0.68 |  | 0.23 (-0.77, 1.22) | 0.65 |
| DBP (mm/Hg) | 0.32 (-0.22, 0.87) | 0.25 |  | 0.27 (-0.28, 0.82) | 0.34 |  | 0.27 (-0.28, 0.82) | 0.34 |  | 0.28 (-0.27, 0.82) | 0.32 |
| Insulin (u/ml) | 1.03 (0.98, 1.07) | 0.25 |  | 1.02 (0.97, 1.06) | 0.43 |  | 1.02 (0.97, 1.06) | 0.45 |  | 1.01 (0.98, 1.05) | 0.45 |
| Glucose (mmol/l)* | 0 (-0.07, 0.07) | 1.00 |  | -0.01 (-0.08, 0.06) | 0.83 |  | -0.01 (-0.07, 0.06) | 0.88 |  | -0.01 (-0.08, 0.06) | 0.82 |
| Triglycerides (mmol/l)* | 1.02 (0.99, 1.05) | 0.21 |  | 1.01 (0.98, 1.05) | 0.37 |  | 1.01 (0.98, 1.04) | 0.41 |  | 1.01 (0.98, 1.04) | 0.56 |
| HDL-c (mmol/l) | -0.01 (-0.03, 0.01) | 0.32 |  | -0.01 (-0.03, 0.02) | 0.64 |  | 0 (-0.03, 0.02) | 0.68 |  | 0 (-0.03, 0.02) | 0.66 |
| LDL-c (mmol/l) | -0.03 (-0.09, 0.03) | 0.28 |  | -0.03 (-0.09, 0.03) | 0.38 |  | -0.02 (-0.08, 0.03) | 0.42 |  | -0.02 (-0.08, 0.04) | 0.46 |
| CRP (mmol/l)* | 1.03 (0.97, 1.08) | 0.33 |  | 1.01 (0.96, 1.07) | 0.60 |  | 1.01 (0.96, 1.07) | 0.61 |  | 1.01 (0.96, 1.06) | 0.68 |
| CIMT (mm) | 0 (-0.01, 0.01) | 0.69 |  | 0 (-0.01, 0.01) | 0.55 |  | 0 (-0.01, 0.01) | 0.54 |  | 0 (-0.01, 0) | 0.46 |
| Pulse wave velocity (m/s) | -0.14 (-0.49, 0.21) | 0.44 |  | -0.15 (-0.51, 0.2) | 0.40 |  | -0.15 (-0.51, 0.2) | 0.39 |  | -0.16 (-0.51, 0.2) | 0.39 |

SEP – socioeconomic position. BMI-body mass index. SBP – systolic blood pressure. DBP – diastolic blood pressure. HDL-c – high density lipoprotein cholesterol. LDL-c - low density lipoprotein cholesterol. CRP – C reactive protein. CIMT – carotid intima-media thickness.
*Coefficients for insulin, triglycerides and CRP have been back transformed from the natural log and can be interpreted as a ratio of geometric means, (e.g. a coefficient of 1.02 would be interpreted as an average of 2% increase in the outcome per category increase in cumulative psychosocial adversity)

**Table S5. Associations of psychosocial adversity with cardiovascular risk factors by high and low childhood SEP**

|  | **High adult SEP (n=1472)** | **Low adult SEP (n=758)** |  |  |
| --- | --- | --- | --- | --- |
|  | **Adjusted for age, sex and childhood SEP** | | |  |
|  | **B (95% CI)** | **B (95% CI)** | **P for interaction** |  |
| BMI (kg/m^2^) | -0.04 (-0.38, 0.29) | 0.1 (-0.36, 0.57) | 0.91 |  |
| Waist circumference (cm) | -0.15 (-1, 0.69) | 0.63 (-0.57, 1.82) | 0.70 |  |
| SBP (mm/Hg) | -0.18 (-1.46, 1.1) | 0.94 (-0.7, 2.58) | 0.25 |  |
| DBP (mm/Hg) | -0.02 (-0.71, 0.68) | 0.79 (-0.13, 1.7) | 0.22 |  |
| Insulin (u/ml) | 1.01 (0.96, 1.07) | 1.02 (0.96, 1.09) | 0.27 |  |
| Glucose (mmol/l)* | -0.03 (-0.12, 0.06) | 0.04 (-0.08, 0.15) | 0.21 |  |
| Triglycerides (mmol/l)* | 1.01 (0.97, 1.05) | 1.02 (0.97, 1.08) | 0.06 |  |
| HDL-c (mmol/l) | -0.01 (-0.03, 0.02) | 0 (-0.04, 0.03) | 0.86 |  |
| LDL-c (mmol/l) | 0.01 (-0.07, 0.08) | -0.08 (-0.18, 0.02) | 0.41 |  |
| CRP (mmol/l)* | 1.03 (0.97, 1.1) | 0.98 (0.9, 1.07) | 0.32 |  |
| CIMT (mm) | 0 (-0.01, 0.01) | 0 (-0.01, 0.01) | 0.65 |  |
| Pulse wave velocity (m/s) | -0.21 (-0.69, 0.28) | -0.06 (-0.56, 0.44) | 0.93 |  |

SEP – socioeconomic position. BMI-body mass index. SBP – systolic blood pressure. DBP – diastolic blood pressure. HDL-c – high density lipoprotein cholesterol. LDL-c - low density lipoprotein cholesterol. CRP – C reactive protein. CIMT – carotid intima-media thickness.
*Coefficients for insulin, triglycerides and CRP have been back transformed from the natural log and can be interpreted as a ratio of geometric means, (e.g. a coefficient of 1.02 would be interpreted as an average of 2% increase in the outcome per category increase in cumulative psychosocial adversity)

**Table S6. Associations of individual types of psychosocial adversity with cardiovascular risk factors (continued on following page)**

|  | **Maltreatment** | |  | **Low maternal bonding** | |  | **Parental mental illness** | | **Parental absence from household** | |
| --- | --- | --- | --- | --- | --- | --- | --- | --- | --- | --- |
|  | **B (95% CI)** | **P** |  | **B (95% CI)** | **P** |  | **B (95% CI)** | **P** | **B (95% CI)** | **P** |
| BMI (kg/m^2^) | -0.22 (-1.15, 0.71) | 0.64 |  | -0.19 (-0.75, 0.38) | 0.52 |  | 0.85 (-0.5, 2.21) | 0.22 | -1.16 (-2.6, 0.28) | 0.11 |
| Waist circumference (cm) | 0.1 (-2.27, 2.47) | 0.94 |  | -0.24 (-1.61, 1.13) | 0.73 |  | 0.72 (-2.74, 4.19) | 0.68 | -2.8 (-6.47, 0.87) | 0.13 |
| SBP (mm/Hg) | 0.11 (-3.49, 3.71) | 0.95 |  | -0.05 (-2.15, 2.06) | 0.97 |  | 0.69 (-4.49, 5.87) | 0.79 | -1.12 (-6.71, 4.47) | 0.70 |
| DBP (mm/Hg) | 0.71 (-1.23, 2.64) | 0.48 |  | 0.3 (-0.82, 1.42) | 0.60 |  | -0.35 (-3.18, 2.48) | 0.81 | 0.49 (-2.53, 3.51) | 0.75 |
| Insulin (u/ml) | 0.99 (0.88, 1.11) | 0.86 |  | 1.06 (0.98, 1.14) | 0.15 |  | 1.07 (0.88, 1.32) | 0.49 | 0.94 (0.75, 1.18) | 0.58 |
| Glucose (mmol/l)* | -0.17 (-0.4, 0.06) | 0.14 |  | 0.08 (-0.06, 0.22) | 0.26 |  | 0.09 (-0.27, 0.45) | 0.62 | -0.14 (-0.51, 0.23) | 0.47 |
| Triglycerides (mmol/l)* | 0.95 (0.86, 1.05) | 0.33 |  | 1.04 (0.98, 1.11) | 0.17 |  | 0.94 (0.81, 1.1) | 0.44 | 1 (0.85, 1.18) | 0.99 |
| HDL-c (mmol/l) | 0.06 (-0.02, 0.14) | 0.14 |  | 0 (-0.05, 0.04) | 0.92 |  | 0.07 (-0.04, 0.18) | 0.24 | -0.01 (-0.13, 0.11) | 0.88 |
| LDL-c (mmol/l) | -0.01 (-0.21, 0.18) | 0.88 |  | 0.03 (-0.09, 0.15) | 0.64 |  | -0.38 (-0.68, -0.07) | 0.02 | -0.09 (-0.41, 0.24) | 0.61 |
| CRP (mmol/l)* | 0.93 (0.78, 1.12) | 0.46 |  | 1.02 (0.91, 1.14) | 0.75 |  | 0.83 (0.64, 1.08) | 0.16 | 1.08 (0.81, 1.44) | 0.59 |
| CIMT (mm) | -0.01 (-0.04, 0.01) | 0.28 |  | 0 (-0.02, 0.02) | 0.98 |  | -0.02 (-0.06, 0.02) | 0.34 | -0.02 (-0.06, 0.02) | 0.40 |
| Pulse wave velocity (m/s) | -0.41 (-1.63, 0.82) | 0.51 |  | -0.41 (-1.18, 0.37) | 0.31 |  | -0.48 (-2.3, 1.35) | 0.61 | 1.88 (-0.1, 3.86) | 0.06 |

BMI-body mass index. SBP – systolic blood pressure. DBP – diastolic blood pressure. HDL-c – high density lipoprotein cholesterol. LDL-c - low density lipoprotein cholesterol. CRP – C reactive protein. CIMT – carotid intima-media thickness.
Results are adjusted for age, sex and childhood socioeconomic position.
*Coefficients for insulin, triglycerides and CRP have been back transformed from the natural log and can be interpreted as a ratio of geometric means, (e.g. a coefficient of 1.02 would be interpreted as an average of 2% increase in the outcome per category increase in cumulative psychosocial adversity)

**Table S6 continued**

|  | **Parental separation or divorce** | |  | **Parental physical illness or disability** | |  | **Parental death** | |
| --- | --- | --- | --- | --- | --- | --- | --- | --- |
|  | **B (95% CI)** | **P** |  | **B (95% CI)** | **P** |  | **B (95% CI)** | **P** |
| BMI (kg/m^2^) | 0.16 (-0.66, 0.98) | 0.70 |  | 0.21 (-0.26, 0.67) | 0.38 |  | 0.02 (-0.72, 0.75) | 0.96 |
| Waist circumference (cm) | 0.28 (-1.81, 2.37) | 0.79 |  | 1.08 (-0.1, 2.26) | 0.07 |  | -0.99 (-2.86, 0.88) | 0.30 |
| SBP (mm/Hg) | -0.24 (-3.36, 2.89) | 0.88 |  | -0.56 (-2.33, 1.21) | 0.54 |  | 3.2 (0.41, 6) | 0.02 |
| DBP (mm/Hg) | -0.1 (-1.8, 1.6) | 0.91 |  | -0.13 (-1.1, 0.83) | 0.79 |  | 1.53 (0, 3.06) | 0.05 |
| Insulin (u/ml) | 1.03 (0.91, 1.16) | 0.62 |  | 1.01 (0.94, 1.09) | 0.75 |  | 0.98 (0.87, 1.09) | 0.69 |
| Glucose (mmol/l)* | -0.02 (-0.23, 0.19) | 0.85 |  | -0.02 (-0.14, 0.11) | 0.78 |  | -0.06 (-0.25, 0.14) | 0.56 |
| Triglycerides (mmol/l)* | 1.03 (0.93, 1.13) | 0.57 |  | 1.02 (0.97, 1.07) | 0.48 |  | 1.02 (0.93, 1.1) | 0.71 |
| HDL-c (mmol/l) | -0.02 (-0.09, 0.05) | 0.65 |  | -0.02 (-0.06, 0.02) | 0.27 |  | -0.03 (-0.09, 0.03) | 0.40 |
| LDL-c (mmol/l) | 0 (-0.19, 0.19) | 0.98 |  | -0.06 (-0.17, 0.04) | 0.23 |  | 0.07 (-0.09, 0.24) | 0.38 |
| CRP (mmol/l)* | 1.04 (0.88, 1.22) | 0.66 |  | 1.08 (0.99, 1.18) | 0.08 |  | 0.94 (0.81, 1.08) | 0.39 |
| CIMT (mm) | 0 (-0.03, 0.02) | 0.94 |  | 0 (-0.01, 0.02) | 0.63 |  | -0.01 (-0.03, 0.02) | 0.54 |
| Pulse wave velocity (m/s) | -0.24 (-1.35, 0.88) | 0.68 |  | -0.2 (-0.82, 0.41) | 0.52 |  | 0.23 (-0.76, 1.23) | 0.65 |

**Table S7. Associations of cumulative psychosocial adversity in childhood and CVD risk factors in complete case samples (i.e. with no missing data) (n=569)**

|  | **Adjusted for age, sex and childhood SEP** | |  |
| --- | --- | --- | --- |
|  | **B (95% CI)** | **P** |  |
| BMI (kg/m^2^) | 0.5 (-0.01, 1.01) | 0.05 |  |
| Waist circumference (cm) | 1.06 (-0.28, 2.39) | 0.12 |  |
| SBP (mm/Hg) | 1.24 (-0.8, 3.27) | 0.23 |  |
| DBP (mm/Hg) | -0.19 (-1.29, 0.92) | 0.74 |  |
| Insulin (u/ml) | 1.03 (0.96, 1.1) | 0.46 |  |
| Glucose (mmol/l)* | -0.01 (-0.15, 0.13) | 0.88 |  |
| Triglycerides (mmol/l)* | 1.05 (0.99, 1.11) | 0.10 |  |
| HDL-c (mmol/l) | 0 (-0.04, 0.04) | 0.96 |  |
| LDL-c (mmol/l) | -0.03 (-0.15, 0.08) | 0.58 |  |
| CRP (mmol/l)* | 1.06 (0.96, 1.18) | 0.24 |  |
| CIMT (mm) | 0 (-0.02, 0.01) | 0.52 |  |
| Pulse wave velocity (m/s) | -1.02 (-2.1, 0.06) | 0.06 |  |

SEP – socioeconomic position. BMI-body mass index. SBP – systolic blood pressure. DBP – diastolic blood pressure. HDL-c – high density lipoprotein cholesterol. LDL-c - low density lipoprotein cholesterol. CRP – C reactive protein. CIMT – carotid intima-media thickness.
*Coefficients for insulin, triglycerides and CRP have been back transformed from the natural log and can be interpreted as a ratio of geometric means, (e.g. a coefficient of 1.02 would be interpreted as an average of 2% increase in the outcome per category increase in cumulative psychosocial adversity)

**Table S8. Associations of cumulative psychosocial adversity in childhood and CVD risk factors after additional adjustment for medication use**

|  | **Adjusted for age, sex, childhood SEP and medication use** | |  |
| --- | --- | --- | --- |
|  | **B (95% CI)** | **P** |  |
| SBP (mm/Hg) | 0.18 (-0.82, 1.18) | 0.72 |  |
| DBP (mm/Hg) | 0.26 (-0.29, 0.81) | 0.36 |  |
| Insulin (u/ml) | 1.02 (0.98, 1.06) | 0.40 |  |
| Glucose (mmol/l)* | 0 (-0.07, 0.07) | 1.00 |  |
| Triglycerides (mmol/l)* | 1.01 (0.98, 1.05) | 0.35 |  |
| HDL-c (mmol/l) | -0.01 (-0.03, 0.02) | 0.60 |  |
| LDL-c (mmol/l) | -0.03 (-0.08, 0.03) | 0.34 |  |

SEP – socioeconomic position. SBP – systolic blood pressure. DBP – diastolic blood pressure. HDL-c – high density lipoprotein cholesterol. LDL-c - low density lipoprotein cholesterol. Where SBP and DBP are the outcomes, associations are adjusted for use of antihypertensive medication. Where insulin and glucose are the outcomes, associations are adjusted for diabetes medication use. When triglycerides, HDL and LDL are the outcomes, associations are adjusted for stain use.

*Coefficients for insulin and triglycerides have been back transformed from the natural log and can be interpreted as a ratio of geometric means, (e.g. a coefficient of 1.02 would be interpreted as an average of 2% increase in the outcome per category increase in cumulative psychosocial adversity)

**Table S9. Associations of cumulative psychosocial adversity in childhood and CVD risk factors after removing participants who reported medication use at the time of outcome assessment (i.e. reported taking any antihypertensive, diabetes or statin medication)**

|  | **Adjusted for age, sex and childhood SEP and medication use** | |  |
| --- | --- | --- | --- |
|  | **B (95% CI)** | **P** |  |
| SBP (mm/Hg) | 0.23 (-1, 1.45) | 0.72 |  |
| DBP (mm/Hg) | 0.32 (-0.36, 0.99) | 0.36 |  |
| Insulin (u/ml) | 1.02 (0.97, 1.06) | 0.48 |  |
| Glucose (mmol/l)* | -0.01 (-0.07, 0.05) | 0.84 |  |
| Triglycerides (mmol/l)* | 1.02 (0.98, 1.05) | 0.28 |  |
| HDL-c (mmol/l) | -0.01 (-0.03, 0.02) | 0.53 |  |
| LDL-c (mmol/l) | -0.03 (-0.09, 0.03) | 0.37 |  |

SBP – systolic blood pressure. DBP – diastolic blood pressure. HDL-c – high density lipoprotein cholesterol. LDL-c - low density lipoprotein cholesterol.

*Coefficients for insulin and triglycerides have been back transformed from the natural log and can be interpreted as a ratio of geometric means, (e.g. a coefficient of 1.02 would be interpreted as an average of 2% increase in the outcome per category increase in cumulative psychosocial adversity).

**References**

1. Charakida M, Khan T, Johnson W, Finer N, Woodside J, Whincup PH, et al. Lifelong patterns of BMI and cardiovascular phenotype in individuals aged 60-64 years in the 1946 British birth cohort study: an epidemiological study. Lancet Diabetes Endocrinol. 2014;2(8):648-54.

2. Steiger JL, JC. Statistically based tests for the number of common factors. Annual Spring Meeting of the Psychometric Society; Iowa City, IA1980.

3. Bentler PM. Comparative fit indexes in structural models. Psychol Bull. 1990;107(2):238-46.

4. Tucker LL, C. A reliability coefficient for maximum likelihood factor analysis. Psychometrika. 1973;38(1):1-10.
